# Glycated hemoglobin level dynamics in COVID-19 survivors: 12 months follow-up study after discharge from hospital

**DOI:** 10.1371/journal.pone.0275381

**Published:** 2022-11-09

**Authors:** Marina Shestakova, Irina Kononenko, Zilya Kalmykovа, Tatyana Markova, Elena Kaplun, Mar’yana Lysenko, Natalya Mokrysheva

**Affiliations:** 1 Endocrinology Research Centre, Moscow, Russia; 2 City Clinical Hospital № 52, Moscow, Russia; 3 Pirogov Russian National Research Medical University, Moscow, Russia; University of Campania Luigi Vanvitelli: Universita degli Studi della Campania Luigi Vanvitelli, ITALY

## Abstract

**Introduction:**

One of the stages of reproduction of SARS-CoV-2 is the S-protein glycosylation to facilitate penetration into target cells. It has been suggested that SARS-CoV-2 is able to enter erythrocytes, interact with heme and porphyrin, which could influence HbA1c levels. Assessment of HbA1c levels in individuals with acute COVID-19 and after recovery may show clinical relevance of this hypothesis.

**Aim:**

To assess HbA1c levels in patients with COVID-19 in the acute phase and in early (6–8 weeks) and late (52±2 weeks) periods after recovery.

**Materials and methods:**

We conducted a multicenter prospective study, which included patients hospitalized in Endocrinology Research Centre and the City Clinical Hospital № 52" diagnosed with COVID-19, virus identified/ not identified. Patients were divided into three groups according to baseline HbA1c level and the presence or absence of previous history of diabetes previous history of diabetes mellitus (DM): HbA1c ≤ 6.0%, HbA1c > 6.0% and patients with DM. Patients were examined during the acute COVID-19 phase and in early (6–8 weeks) and late (52±2 weeks) periods after recovery. Oral glucose tolerance test was performed in the group with initial HbA1c > 6.0% to clarify the diagnosis.

**Results:**

We included 194 patients in the study. During the follow-up, 52 patients were examined in 6–8 week period: 7 with HbA1c ≤ 6.0%, 34 with HbA1c > 6.0%, 11—with previously diagnosed DM. Carbohydrate metabolism assessment in the later stages (52±2 weeks) after recovery was performed in 78 patients: 33 patients with HbA1c ≤ 6.0%, 36 patients with HbA1c > 6.0% and 9 patients with previously established diabetes. HbA1c median in patients with HbA1c ≤ 6.0% was 5.7% [5.3;5.8], with HbA1c>6.0% -6.4% [6.2; 6.6], with previously diagnosed DM—7.7% [7.2; 8.9]. Statistically significant decrease in HbA1c over time 6–8 weeks after extracts were obtained in both groups of individuals without a history of DM (Wilcoxon test, p<0.05). After 52±2 weeks we observed HbA1c decrease in all three groups (Fridman test, p<0.05): in patients with HbA1c ≤ 6.0% median HbA1c was 5.5[5.3;5.7], with HbA1c>6.0% - 6.1[6.15;6.54], with previously diagnosed DM—7.8 [5.83; 8.08]. Development of DM after 52±2 weeks was recorded in 7.24% of all examined patients without a history of DM, which is 16.6% of the total number of patients examined in dynamics with HbA1c > 6.0%.

**Conclusion:**

HbA1c elevation during the acute phase of COVID-19 may be false due to the effect of SARS-CoV-2 on hemoglobin kinetics and/or detection on the surface of the SARS-CoV-2 virion highly glycosylated S-proteins by high performance liquid chromatography determinations. Upon detection HbA1c > 6.0% in patients with COVID-19 in the active phase of the disease without concomitant hyperglycemia re-determine the level of HbA1c after recovery is recommended.

## Introduction

The impact of SARS-CoV-2 on various cells, organs, and physiology is a matter of extensive discussion. In particular, there is evidence of carbohydrate metabolism (CM) disorders diagnosed for the first time in patients with the new coronavirus infection COVID-19. At the same time, a positive correlation is observed between higher HbA1c levels, inflammatory manifestations, and severity of COVID-19 [1, 2]. However, it is a still an open question whether the identified CM disorders are persistent or transient?

The process of SARS-CoV-2 reproduction involves Spike-protein (S-protein) glycosylation infecting target cells [3, 4]. It is assumed that SARS-CoV-2 infects erythrocytes and captures the porphyrin to inhibit heme metabolism [3, 5], resulting in elevated HbA1c in the acute stage of the disease. HbA1c testing is required during the acute phase of COVID-19 to confirm this hypothesis, with follow-up after virus elimination and patient’s recovery.

### Aim of the study

To assess HbA1c levels in patients with COVID-19 in the acute phase and in early (6–8 weeks) and late (52±2 weeks) periods after recovery.

## Materials and methods

### Design of the study

Multicentre, observational, prospective, observed study. We did not calculate sample for the study.

### Inclusion criteria

Patients admitted to hospitals with diagnosis ‘COVID-19, viral pneumonia’.

### Exclusion criteria

No data on HbA1c level at admission.

1. Use of drugs that affect CM (systemic glucocorticoids, etc.) before hospital admission.
2. History of chemotherapy or cancer in the past six months.
3. Medical history of severe renal or liver disorders.
4. Blood diseases.

### Length of research study

The study included patients in the acute phase of COVID-19 admitted to the Endocrinology research centre and City Clinical Hospital № 52 in Moscow from May 5, 2020 to October 2020. In 6–8 weeks after discharge examination were enrolled patients from both hospitals, in 52±2 weeks—only patients treated at the Endocrinology research centre.

## Methods

All the enrolled patients had the following tests performed:

At admission—oropharyngeal and nasopharyngeal RT-PCR for SARS-CoV-2, glycated hemoglobin (HbА1c), admission blood glucose (ABG), chest computed tomography (CCT). complete blood count (indicating RBC count and hemoglobin level), blood chemistry panel (BUN, creatinine, eGFR using CKD-EPI, serum electrolytes, ALT, AST, bilirubin).

On the second day of hospitalization—fasting plasma glucose (FPG).

Anthropometric measurements were obtained for all patients and medical history was taken concerning the prior diagnosis of DM or use of glucose-lowering drugs. Patients were examined and treated by the guidelines ‘Prevention, diagnosis, and treatment of the new coronavirus infection COVID-19’, version 6 (dated 28.04.2020), provided by the Russian Ministry of Health Care.

Laboratory tests were performed in the clinical diagnostic laboratories of the Endocrinology research centre and City Clinical Hospital № 52 in Moscow.

- COVID-19 diagnosis was based on the detection of SARS-CoV-2 RNA by oropharyngeal and nasopharyngeal RT-PCR and/or on the typical chest CT findings of COVID-19 pneumonia—multifocal ground-glass opacity together with specific clinical manifestations (fever, weakness, loss of smell).
- HbA1c was measured by high-performance liquid chromatography on Bio-Rad D10 –an NGSP-certified method (the National Glycohemoglobin Standardisation Program). The HbA1c ≤ 6.0% is considered a normal value.
- Blood chemistry was performed on Architect c8000 clinical chemistry analyzer (Abbott Laboratories, USA) according to standard assay protocols. Reference ranges are as follows: glucose 3.1–6.1 mmol/L, ALT– 0.0–55.0 U/L, AST– 5.0–34.0 U/L, creatinine– 50–98 *μ*mol/L, potassium– 3.5–5.1 mmol/L, sodium– 136–145 mmol/L, LDH- 125–220 U/L, albumin—34–48 g/L.
- CCT data were obtained using Aquilion One (Toshiba Medical Systems Corporation, Japan) scanner; Thoracic VCAR software integrated with AW Server 3.2. workstation by General Electric was used for CT image analysis.
- Estimated average glucose (eAG) was calculated based on HbA1c level by the formula: eAG(mmol/L) = 1.5944 x HbA1c (%)– 2.594 [6].
- Stress hyperglycemia is defined by ADA (American Diabetes Association) as an elevation of fasting glucose ≥ 7 mmol/L, or 2-hour postprandial glucose ≥ 11 mmol/L, in a patient without evidence of previous diabetes [7]

### Ethical statement

A local ethics committee of the National Medical Research Centre for Endocrinology approved the study protocol no. 124 dated 5^th^ May 2020. All enrolled patients signed informed consent documentation before inclusion.

### Subjects

194 patients satisfied the inclusion criteria. Patients were split into 3 groups based on the history of CM disorders and HbA1c: **group A** included patients without DM history with HbA1c ≤ 6.0%; **group B** included patients without DM history with HbA1c>6.0%; **group C** included patients with DM history. We considered HbA1c ≤ 6.0% to be within the normal range specified in the algorithms of specialized medical care for patients with DM [8]. HbA1c >6.5% was used as a diagnostic criterion for DM [9]. On 6–8 weeks of follow-up 52 patients were included.

### Statistical analysis

Statistical analysis was performed using StatSoft© STATISTICA 13.3.0 (TIBCO Software Inc., USA), MS Excel 14.7.2 update and Wizard 1.9.42 (267) update software. The Kolmogorov-Smirnov test was used to determine the distribution. Data were reported as median values (IQR) [25; 75] or proportions (%). The Mann-Whitney U test was used to compare parameters in study groups, and the Kruskal Wallis test was used to compare values across groups. The Wilcoxon signed-rank test was used to compare dynamic values across matched samples. Differences were deemed statistically significant at p-value <0.05.

## Results

Of 234 patients admitted to the hospitals, 194 met the criteria and were included in the study. Patients were hospitalized on the 6 [4;9] day from the first symptoms due to progressive disease. The early disease onset in all patients was characterized by fever (38°-39°C), most patients receiving antipyretic therapy before hospitalization (paracetamol up to 4 times a day maximum). Median age 58 years [47; 71.5], male/female ratio (%) 48.6/51.4, BMI 29.8 [25.3; 33.2] kg/m^2^. Concomitant diseases: 22.8% of patients had obesity stage 1–3, 43.9% of hospitalized patients had arterial hypertension, 5.7%—coronary artery disease, while 7 (5.47%) patients had a previous heart attack, 5 (3.3%) had coronary revascularization, 6 (4.0%) patients had a stroke, lung diseases (COPD, bronchial asthma, etc.) - 13 (8.7%) patients. All patients were stable. Patients did not need oxygen support, median SpO2 97% [93; 99].

**Fig 1:**
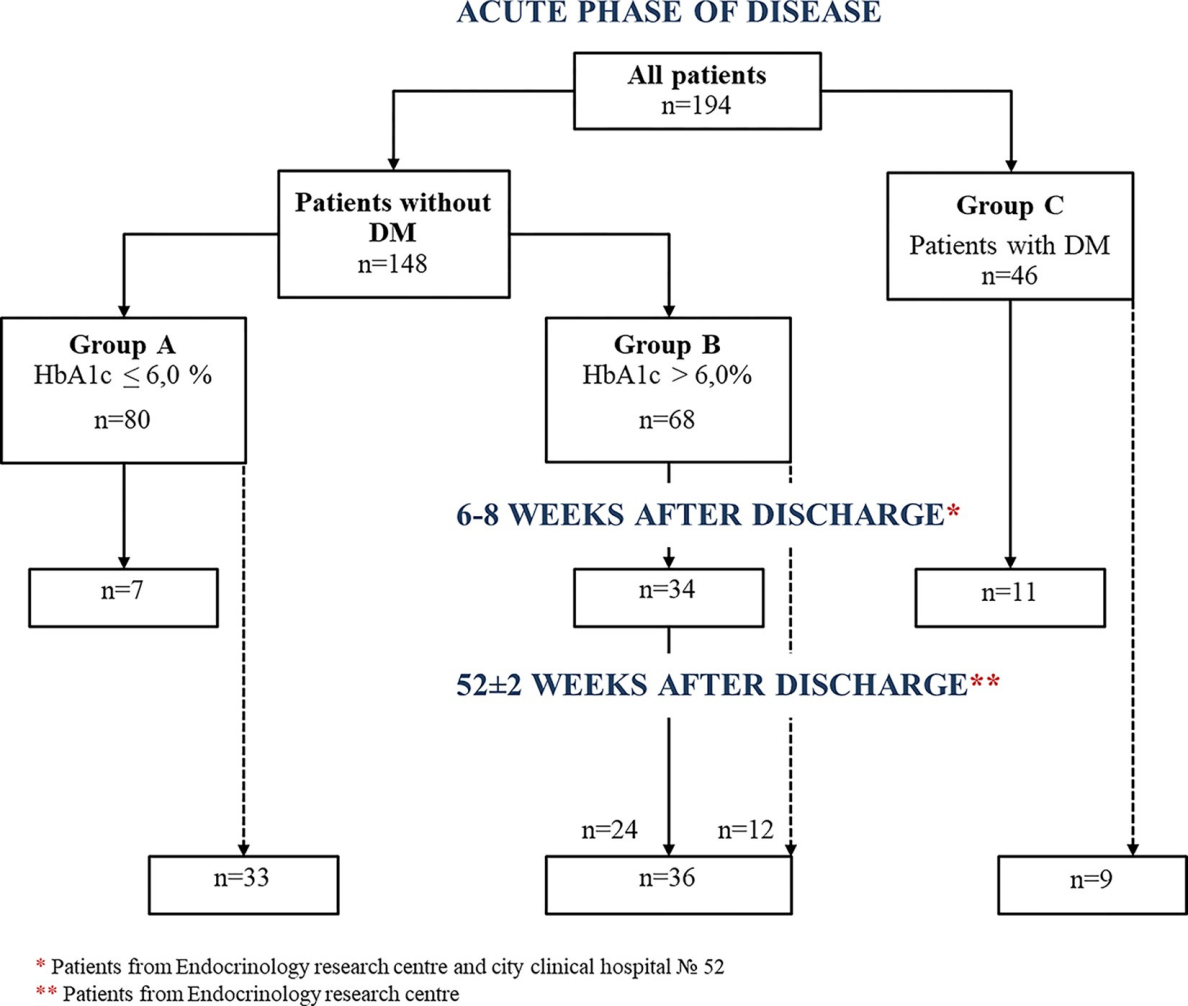
Study design. DM, diabetes mellitus; HbA1c, glycated hemoglobin.

Group A included 80 patients (41,2%), group B– 68 (35%), group C—46 (23,7%) (Fig 1).

Group C included 45 patients with T2DM and 1 with T1DM, the duration of diabetes was 4–23 years. Characteristics of antidiabetic therapy before admission, during hospitalization and at the time of discharge are presented in Table 1.

**Table 1:** Characteristics of antidiabetic drugs in patients with DM history (n = 46).

Characteristics	At admission	During in-hospital stay	On discharge
T2DM, n (%)	45	**-**	**-**
T1DM, n (%)	1	**-**	**-**
Insulin use, n (%)	8 (17.3)	34 (73.9)	30 (65.2)
Sulphonyl urea, n (%)	2 (4.3)	-	-
Biguanides, n (%)	31 (67.3)	-	-
DPP-4 inhibitors, n (%)	21 (45.6)	12 (26.0)	12 (26.0)
GLP-1 receptor agonist, n (%)	8 (17.3)	-	-
SGLT-2 inhibitors, n (%)	13 (28.2)	-	-
No treatment	4 (8.7)	-	-

Data are presented as means ± SD.

T2DM, type 2 diabetes mellitus; T1DM, type 1 diabetes mellitus; DPP-4, dipeptidyl peptidase 4; GLP-1, glucagon-like peptide 1; SGLT-2, sodium glucose co-transporter-2

### Assessment of HbA1c in the acute phase of COVID-19

Before admission, DM was diagnosed in 46 (23.5%) patients: T2DM in 43 and T1DM in 3 patients, DM duration 4–23 y, median HbA1c 7.7% [7.2; 8.9] (Table 2).

**Table 2:** Clinical characteristics and CM values in hospitalized patients with COVID-19, depending on baseline HbА1c and DM history.

Parameters	Patients w/o DM history		Patients with known DM Group C (n = 46)	Kruskal Wallis test, Mann-Whitney test, *p-value*
	HbA1c ≤ 6,0% Group A (n = 80)	HbA1c ≥ 6,0% Group B (n = 68)		
HbA1c at admission, %	5,7 [5,3; 5,8]	6,4[6,2; 6,6]	7,7 [7,2; 8,9]	**<0,001** p_1-2_ **= 0,003** p_1-3_**<0,001** p_2-3_ = 0,002
Gender, no. of patients (%) M/F	46/25 65%/35%	34/44 43,5%/56,5%	17/29 37%/63%	0,019
Age, y	50 [41;59]	60,5 [52;74,5]	62 [54;70]	**<0,001** p_1-2_ **= 0,003** p_1-3_ **= 0,002** p_2-3_ = 0,126
BMI kg/m^2^	27,3 [24,6;30,5]	29,8[25;33,9]	30,9 [28,58;36,36]	0,087
Hemoglobin, g/L	143[132,2;149,7]	132[123;143,7]	133,5 [126,5;145,5]	0,113
GFR by EPI, mL/min/1,73m^2^	83,2[69,0;102,5]	75,8[56,6;96,7]	74,17[49,7;90,7]	0,231
FPG, mmol/L	5,03[4,9;5,33]	5,33[5,0;5,9]	8,2[7,0;10,6]	**<0,001** p_1-2_ **= 0,131** p_1-3_**<0,001** p_2-3_**<0,001**
ABG, mmol/L	5,78[5,39;6,3]	6,39[5,85;7,02]	10,34[7,47;12,84]	**<0,001** p_1-2_ **= 0,076** p_1-3_**<0,001** p_2-3_ **<0,001**
eAG, mmol/L	6,49[6,21;6,65]	7,61[7,29;7,76]	9,68[8,92;11,63]	**<0,05** p_1-2_ **= 0,064** p_1-3_ **= 0,004** p_2-3_ = **0,016**

The data is presented as median value (Ме) and interquartile range [25; 75] or mass percentage (%).

HbA1c, glycated hemoglobin; BMI, body mass index; GFR, glomerular filtration rate; FPG, fasting plasma glucose, mmol/L; 24hMPG, 24-hour mean plasma glucose over the last three months; SIH, stress-induced hyperglycemia, mmol/L; ABG, admission blood glucose, mmol/L.

Among 148 patients (76.5%) without previous DM 80 (41.2%) had HbA1c ≤ 6.0%. In all patients **with HbA1c ≤ 6.0%** FPG and ABG values did not meet DM diagnostic criteria. Median HbA1c was 5.7% [5.3;5.8]. **68 (35.0%) patients** without DM history had **HbA1c>6.0%, 19 of them had HbA1c ≥6.5%.** Median HbA1c in that group was 6.4% [6.2; 6.6]. In the group with HbA1c ≥ 6.5%, elevated FPG ≥7.0 mmol/L and/or ABG ≥11.1 mmol/L was noted in 9 (5.85%) patients–T2DM was diagnosed. Median age of these patients was 60.5 [52;74.5]. A lifestyle modification and anti-diabetic treatment were initiated. Patients with newly diagnosed DM were excluded from follow-up. Abnormal fasting glycemia (FPG ≥6.1 mmol/L, but <7.0 mmol/L) was detected in 13 patients with HbA1c>6.0%, ABG >7.8 mmol/L was detected in 4 patients. No cases of stress hyperglycemia were observed in our patients.

Glucocorticosteroids (GC) were used in 48 patients (21.1%) (Table 3) only after the assessment of FBG, ABG and HbA1c to avoid possible effect on CM. No significant correlations were identified between GC dose and HbA1c levels.

**Table 3:** GC medication in COVID-19 patients.

Parameters	Patients w/o DM history		Patients with known DM Group C (n = 46)
	HbA1c ≤6,0% Group A (n = 33)	HbA1c≥ 6,0% Group B (n = 36)	
GC medication, no. of patients (%)	10 (21,7%)	27(35%)	27(35%)
Dexamethasone/ Methylprednisolone, no. of patients (%)	8(2%)	15(12%)	11 (23,9%)
24-hour mean dose of oral dexamethasone, mg (max, min)	11,1 (2;16)	10,4 (4;16)	10 (6;15)
24-hour mean dose of oral and/or iv methylprednisolone, mg (max, min)	61,7 (45;80)	117,1 (30;250)	15,5 (12;16)
Duration of treatment, days	7	15	125 (1 patient)

Data are presented as means ± SD.

GC, glucocorticoids; DM, diabetes mellitus; HbA1c, glycated hemoglobin А1с.

HbA1c level was assessed 6–8 weeks after discharge in patients with FPG and ABG values below the diagnostic values for DM.

**Fig 2:**
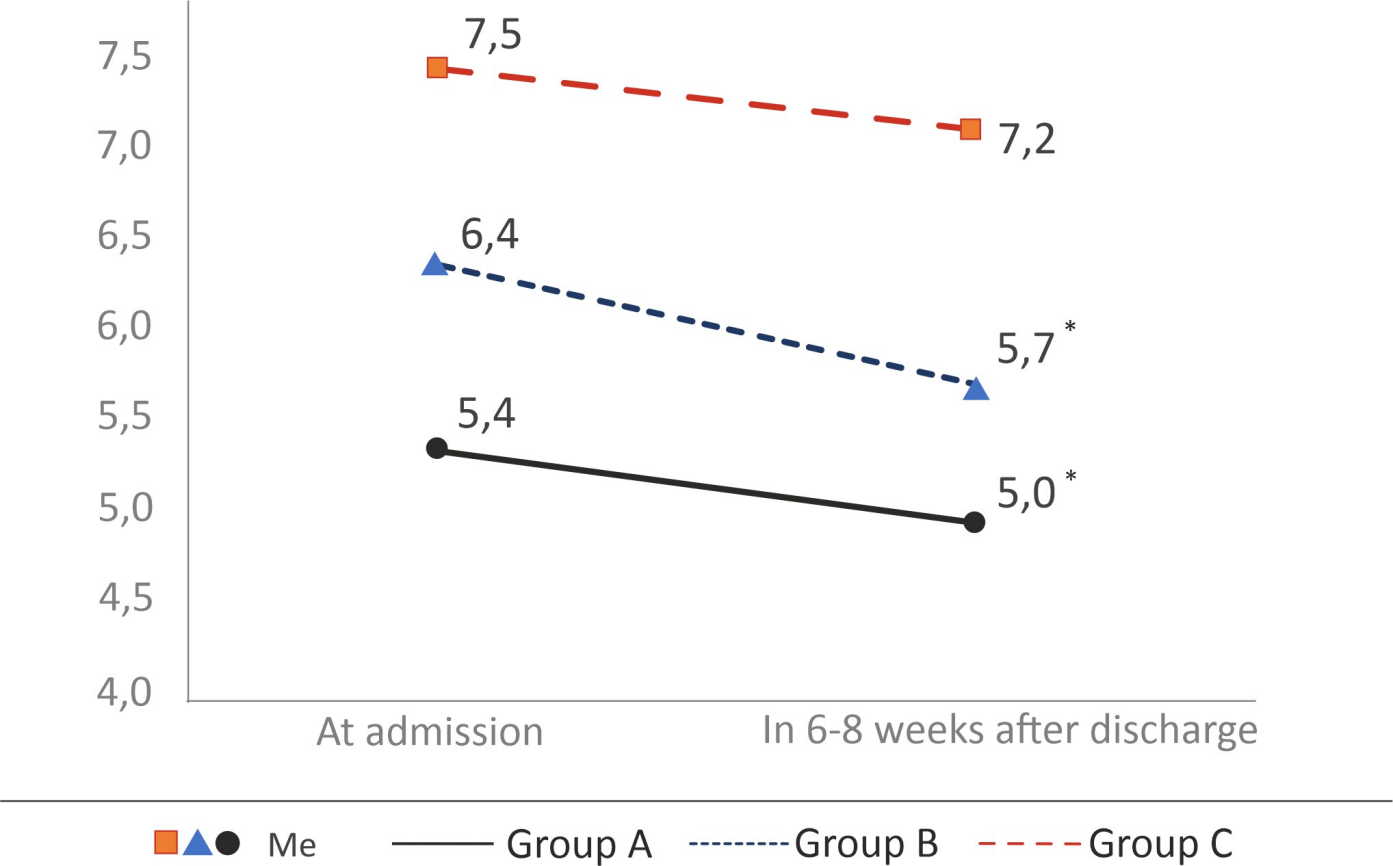
HbA1c dynamics in patient groups in 6–8 weeks after discharge. Me–median.

Overall, HbA1c was measured in the acute phase of COVID-19 and 6–8 weeks after discharge in 52 patients: 7 patients with HbA1c ≤ 6.0% (group A); 34 patients with HbA1c> 6.0% (group B); 11 patients with known DM (group C). In all patients FPG and ABG levels did not exceed DM diagnostic values. A decrease in HbA1c was observed in all groups. In patients with HbA1c ≤ 6.0% median HbA1c was 5.0[4.8;5.3], with HbA1c>6.0% - 5.7[5.5;5.9], with previously diagnosed DM—7.2 [5.6; 9.4] (Fig 2).

In 3 patients HbA1c remained unchanged, insignificant HbA1c elevation (by less 0.5% of baseline) was observed in 2 patients from group C and 2 patients from group A. In group B 3 patients with HbA1c≥6.5% were diagnosed with DM based on WHO criteria, following additional examination (OGTT after same 6–8 weeks), recommendations for treatment and nutrition were given.

Statistically significant differences in HbA1c over observed periods were found in groups A,B (p<0.05). Changes in HbA1c for group C were not statistically significant (p = 0.124).

**Fig 3:**
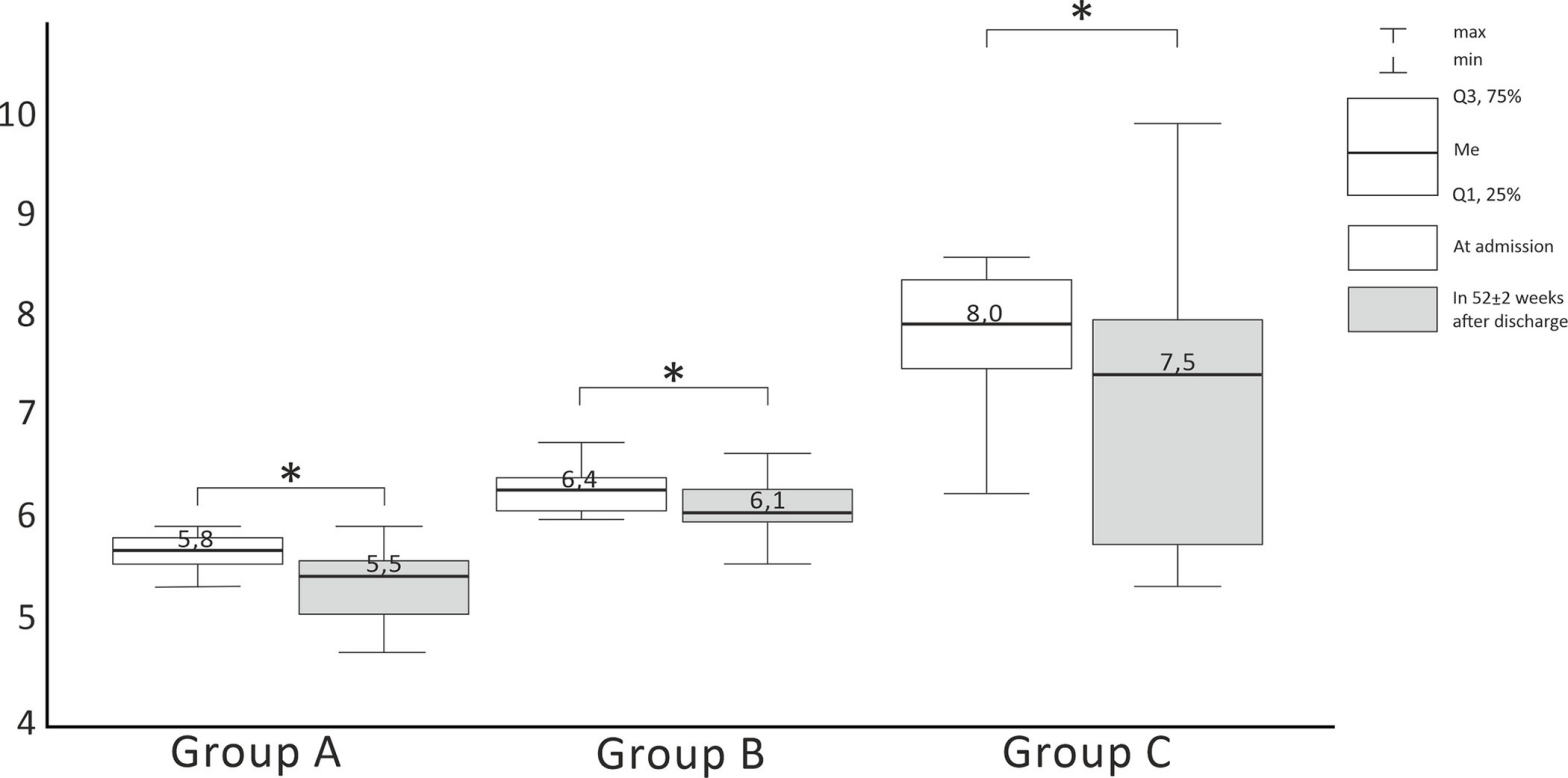
HbA1c dynamics in patient groups at week 52±2. Me–median.

CM was assessed at 52 + 2 weeks after recovery in 78 patients: 33 patients with HbA1c ≤ 6.0% (group A), 36 patients with HbA1c> 6.0% (group B), and 9 patients from group C. Table 4 summarizes clinical and CM parameters in patients examined 52±2 weeks after discharge. We observed significant differences in HbA1c levels across s at admission and 52±2 weeks after discharge (the Kruskal Wallis H test, p<0.001) (Fig 3).

**Table 4:** Clinical and carbohydrate metabolism parameters in patients examined 52±2 weeks after discharge.

Parameters	Patients w/o DM history		Patients with known DM Group C (n = 9)	Kruskal Wallis test, Mann-Whitney test, *p-value*
	HbA1c ≤ 6,0% Group A (n = 33)	HbA1c ≥ 6,0% Group B (n = 36)		
Gender, no. of patients (%) M/F	20/13 60,6%/39,4%	12/24 33,4%/66,6%	3/6 33,3%/66,7%	0,156
Age, y	48,5 [42;56,5]	62 [54;70]	62 [54;70]	0,111
BMI kg/m^2^	27,5 [24,5;30,6]	30,2[26,05;33,55]	29,55 [27,05;33,3]	0,076
HbA1c at admission, %	5,8 [5,6; 5,9]	6,4[6,2;6,5]	8,2 [7,6; 8,5]	**<0,001** p_1-2_ = 0, 013 p_1-3_**<0,001** p_2-3_**<0,001**
HbA1c at 52±2 weeks, %	5,5[5,3;5,7]	6,1[5,9; 6,4]	7,8 [5,9; 8,1]	**<0,001** p_1-2_ = 0,031 p_1-3_**<0,001** p_2-3_ **= 0,012**
FPG at admission, mmol/L	5,15[4,91;5,45]	5,38 [5,02;6,06]	8,88[7,94;12]	**<0,05** p_1-2_ = 0,123 p_1-3_ **= 0,009** p_2-3_ **= 0,012**
FPG at 52±2 weeks, mmol/L	5,27[5,08;5,71]	5,33[4,86;5,74]	5,95[5,57;9,79]	0,254

The data is presented as median value and quartile range (Me [Q25; Q75]). The Kruskal Wallis test was used to compare groups.

HbA1c, glycated hemoglobin; BMI, body mass index; FPG, fasting plasma glucose, mmol/L.

The Wilcoxon signed-rank test was used to compare HbA1c dynamics at week 52±2 across groups (Table 5*)*. Statistical analysis of CM at weeks 6–8 was performed for group B patients only. One year after discharge, a significant decrease in HbA1c was noted in all groups (p<0.05). In group A HbA1c decrease was detected in 81.2% patients, in 9.3% HbA1c remained unchanged, 9.3% showed insignificant HbA1c elevation by <0.5% of baseline. Most DM patients (81.8%) demonstrated HbA1c lowering, while insignificant HbA1c elevation by <0.5% was observed in 18.1%.

**Table 5:** HbA1c dynamics in patient groups at week 52±2.

	HbA1c at admission, %	HbA1c 52±2 weeks after discharge, %	Wilcoxon test, *p-*value
**HbA1c ≤ 6,0%** **Group A (n = 33)**	5,8[5,68; 5,9]	5,5[5,3;5,7]	<0,001
**HbA1c > 6,0%** **Group B (n = 24)**	6,4[6,23;6,51]	6,1[6,15;6,54]	<0,05
**Patients with known DM** **Group C (n = 9)**	8,1 [7,53; 8,48]	7,8 [5,83; 8,08]	<0,05

The data is presented as median value (Ме) and interquartile range [25; 75] or mass percentage (%).

DM, diabetes mellitus; HbA1c, glycated hemoglobin; BMI, body mass index.

Of particular interest was in group B with baseline HbA1c>6.0%. This group presented significant HbA1c lowering from 6.4% to 6.1% (р<0.05). In the absence of treatment, 37.5% patients showed HbA1c falling to normal range, in 41.6% HbA1c values stayed in the grey zone, 20.8% (n = 5) had elevated HbA1c ≥ 6.5%. To clarify the diagnosis for the latter of patients, an OGTT was performed. DM was diagnosed according to the WHO criteria only in 2 patients (1 patient with baseline HbA1c ≥ 6.5%, 1 patient with HbA1c 6.1–6.4%). Overall, 52±2 weeks follow-up allowed to diagnose DM in 5 patients (7.24%): in 3 patients at 6–8 weeks after discharge, in 2 at 52±2 weeks. In the acute phase of COVID-19 all these patients demonstrated HbА1c in «grey zone»: >6.0%, but less than 6.5%. None of the patients with normal baseline CM reported elevated HbA1c >6.0% or FPG ≥6.1 mmol/L on 1-year follow-up.

## Discussion

Cellular ACE2 and TMPRSS2 expression are considered as main points of entry for SARS-CoV-2 [10]. Interestingly, microRNAs are also involved in COVID19 pathogenesis, targeting both ACE2 and TMPRSS2 [11, 12]. Total ACE2, glycosylated ACE2, and TMPRSS2 protein expression was shown to be overexpressed in cardiomyocytes from autopsy cardiomyocytes of patients with DM compared with non-DM patients. which could increase the susceptibility to COVID-19 infection in DM patients [13]. ACE2 expression is also found in pancreatic cells–both exocrine glands and islets, which could lead to exocrine [14] and endocrine [15] COVID-19 complications. However, direct clinical implications of these findings are yet to be described in future studies.

Hyperglycemia is a recognized factor of worse prognosis in patients with COVID-19 [16], and patients with T2DM have significantly higher rates of severe disease and need for intensive care unit [17]. Sardu et al. showed increased IL-6 and D-dimer levels in patients with hyperglycemia and suggested insulin infusion as an effective method for glycemic control and improved outcomes in patients with COVID-19 [18]. In our study, we did not compare the course and outcomes of the disease between patients with or without DM, which should be further addressed in prospective studies. In this part of our study, we did not analyze the relationship between the state of carbohydrate metabolism and the markers of COVID-19 severity, nor did we evaluate the effect of COVID-19 therapy on the state of carbohydrate metabolism.

As well as in DM cases, patients with hypertension are considered to have worse COVID-19 prognosis [19]. It is also suggested that SARS-COV-2 could cause endothelium dysfunction and hypercoagulation, which leads to increased rates of adverse cardiac events [20] In our cohort, patients with or without hypertension and with different hypertensive therapies did not differ in severity or outcomes, which could be attributed to a relatively small sample size. We did not observe myocardial infarction, pericarditis, myocarditis, heart failure and cardiac deaths in our study, both during hospitalization and follow-up. A hyperglycemic state, also in non-diabetic subjects, may be associated with acute conditions and outcomes–especially with acute coronary syndrome [21]. Tight glycemic control demonstrated cardioprotective effect—high efficiency in the cardiovascular disease prevention [22], acute strokes [23], and also COVID-19 [24]. At the same time, a number of comorbidities lead to a more severe course of COVID-19, such as renal failure [25] and liver diseases [26].

The study demonstrated elevated HbA1c>6.0% in 35% of patients hospitalized with a new coronavirus infection without previous DM history. HbA1c ≥6.5% at admission was registered in 13.9%. Only in 5.85% patients HbA1c level ≥6.5% was associated with FPG or ABG elevation according to DM diagnostic criteria, suggesting earlier undiagnosed DM. In all other patients, detection of CM disorders in the acute phase of COVID-19 was challenged by mismatches in elevated HbA1c vs. normal FPG and ABG values. A meta-analysis of 8 retrospective cohort clinical studies suggested that the mean occurrence of newly diagnosed DM in hospitalized COVID-19 patients stands at 14.4% [27]. In most studies, DM was diagnosed based on glycemic values. However, the authors emphasize that in 6 studies HbA1c was not detected in all patients, providing no data to differentiate between newly diagnosed DM or stressed glycemia vs. undiagnosed DM. The European Association of Endocrinology and ADA recommend continuous glycemic glucose and HbA1c monitoring in hospitalized patients with newly diagnosed glycemia over 7.8 mmol/L as a differential diagnosis tool between differential diagnosis of SH and previously undiagnosed DM. As a more stable compound, HbA1c is assumed to be less prone to variability than glucose in acute inflammation or surgical intervention; therefore, HbA1c is considered the most appropriate diagnostic marker of CM disorders in hospitalized patients [28]. Patients showing previously unknown HbA1c≥6.5% at hospitalization should be considered earlier undiagnosed DM [28, 29]. Meanwhile, a few studies report that patients hospitalized with COVID-19 often demonstrate elevated HbA1c for the first-time during hospitalization. Z Wang et al. reported HbA1c>6.0% in 33.3% patients of a general therapy department [1]. Another study refers to previously unknown HbA1c≥6.5% in 24.0% of more critical ICU patients [30]. In most cases, elevated HbA1c was associated with hyperglycemia. A prospective study by J. Wexler et al. reported НbА1с>6.1% in 18% of patients in the general emergency department. On 1-year follow-up examination, DM was established in 15% of patients [31].

The previously unknown HbA1c elevation was also described in COVID-19 patients. Most authors interpret HbA1c values ≥6.5% as newly diagnosed T2DM [32, 33]. In our study, 8% of examined patients demonstrated HbA1c≥6.5% and normal ABG, FPG levels. All conditions associated with falsely elevated HbA1c were excluded, i.e., metabolism disorders (triglycerides, bilirubin, iron), anemia, a decline in GFR, excessive intake of specific drug groups, etc. [34].

Increased secretion of endogenous steroids is an important component of the general adaptation to stress, which helps to maintain homeostasis. Severe illnesses, trauma, anesthesia, and surgery are accompanied by activation of the hypothalamic–pituitary–adrenal axis [35].

Increased levels of endogenous steroids may lead to an acute increase in glucose level, even in non-diabetic patients. Single blood glucose test reflects current glucose levels only, whereas the HbA1c test serves as an overall marker of average glucose levels for previous 2–3 months. In our cohort, patients were hospitalized within a week since the disease onset. Moreover, patients had no increase in ABG or FPG, which suggests their HbA1c levels could not be affected by stress hyperglycemia.

In most patients, including those with DM, under dynamic control both after 6–8 weeks and 52±2 weeks after discharge, HbA1c significantly decreased. However, the bodyweight of the examined individuals returned to baseline values within a few months after recovery. All possible reasons for the decrease in HbA1c in 52 ± two weeks after recovery were also excluded: taking CM- modifying drugs, diet, weight loss, etc. The therapy in patients with previous DM did not change compared to the baseline. Meanwhile, newly diagnosed DM was reported during dynamic follow-up after recovery in 5 patients only (7.24%): 4 patients with baseline HbA1c≥ 6.5% and one patient with HbA1c 6.1–6.4%. Two more patients with HbA1c≥6.5% were diagnosed with IGT. Diagnoses were formulated based on WHO recommendations [36, 37]. The results suggest that SARS-CoV-2 may induce a diabetogenic effect and contribute to DM progression in patients with existing CM disorders and high DM risk. However, further studies are needed.

Long-term analysis of the state of CM in patients after COVID-19 and the results of dynamic monitoring of patients with newly diagnosed CM disorders in the acute stage of COVID-19 were not published. Our study is a pioneer attempt to analyze HbA1c dynamics in COVID-19 patients. Our results report transient HbA1c elevation in patients with acute COVID-19 with an unexpectedly fast lowering to normal range in 6–8 weeks. No relevant studies were found to report similar HbA1c changes in other inflammatory diseases as community-acquired pneumonia (CAP) attributed to other causes.

It cannot be ruled out that a transient increase in HbA1c levels during the acute period of COVID-19 may be associated with specific SARS-CoV-2 structure and replication and primary disruption of erythropoiesis According to the results of previous studies, erythrocytes are one of the target cells in COVID-19, which is further proved by potential abnormalities in such forms of hemoglobin, as carboxyhemoglobin, methemoglobin, [38] or hypoxia and SpO2 decrease in many patients.

**Fig 4:**
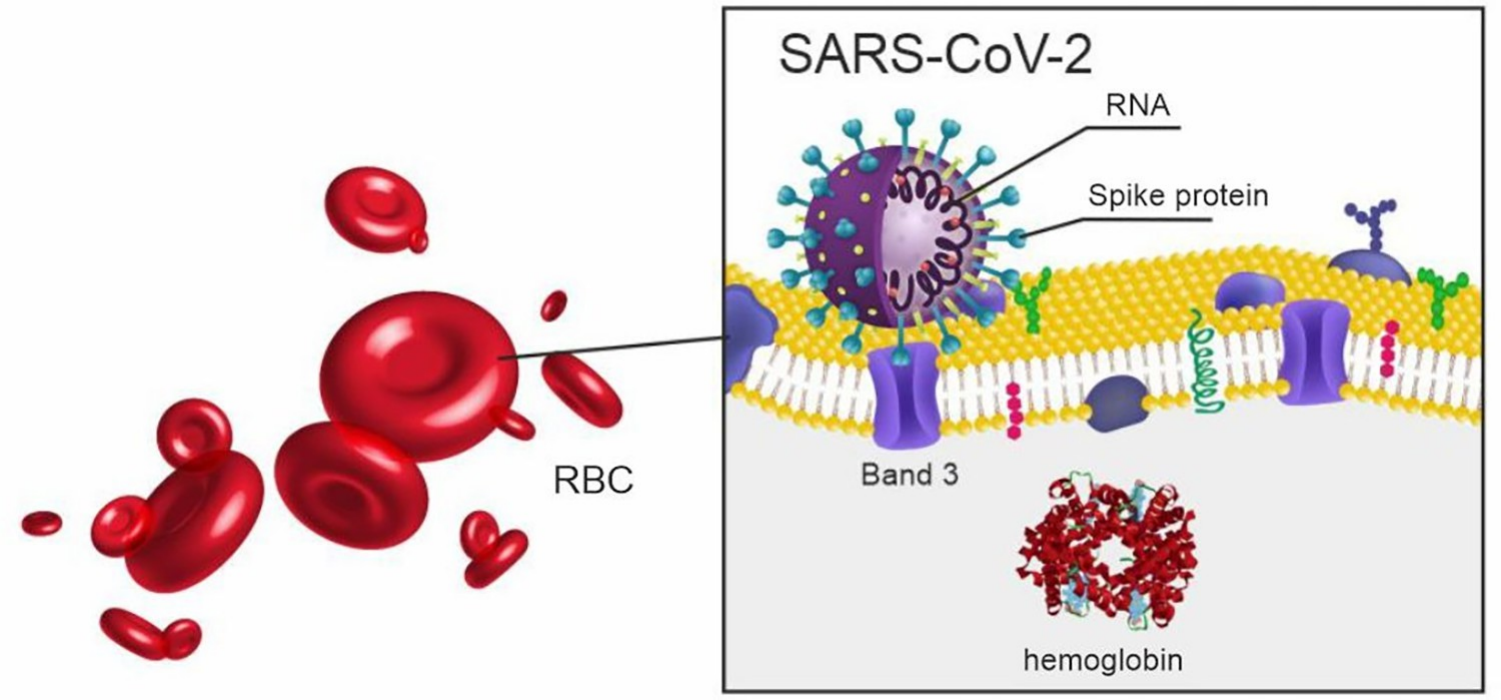
SARS-CoV-2 virus infecting RBC. SARS-CoV-2, severe acute respiratory syndrome-related coronavirus 2; RBC–red blood cell.

I. Cosic et al. l. [5] developed an original biophysical Resonant Recognition Model to demonstrate the SARS-CoV-2 ability to infect RBC through the interaction between the S- protein and erythrocytes Band3 surface proteins (Fig 4). W. Liu et al. described how the virus attacks the 1-beta chain of hemoglobin and captures the porphyrin to inhibit human haem metabolism [3]. Moreover, HbA1c elevation is also associated with thalassemia, characterized by decreased beta-polypeptide chains and faulty hemoglobin synthesis [39].

SARS-CoV-2 virions infect cells through its S-protein of the lipid bilayer. The S-protein binds to specific receptors on the surface of target cells. A group of researchers in China utilized mass spectrometry to investigate the structure and post-translational modifications of SARS-CoV-2 proteins. They demonstrated that the S-protein is densely glycosylated due to extensive N-linked glycosylation and the high content of carbohydrate fragments. It was calculated that carbohydrate chains cover two-thirds of the SARS-CoV-2 virion surface [40]. In the present study, the measurement of HbA1c was performed on a Bio-Rad D10 HPLC analyzer certified by NGSP and currently considered a gold standard HbA1c test. The technology utilizes ion-exchange resins to absorb different forms of hemoglobin and separate different hemoglobin fractions by pushing the molecules across a pН gradient [41]. Considering the structural features of the virus, it is impossible to exclude obtaining falsely elevated HbA1c corresponding to hemoglobin and virion site-specific glycosylation.

## Limitations

The limitations of our study are its relatively small number of hospitalized patients, and lack of information about pre‐infection glycemic control and state of carbohydrate metabolism. We also didn’t explore general COVID-19 treatment and its association with glycemic state. It would be interesting to learn whether HbA1c predicts COVID‐19 mortality and outcomes, need for ICU.

## Conclusion

HbA1c might be falsely elevated in patients with acute stage of COVID-19 as a result of disrupted hemoglobin kinetics caused by SARS-CoV-2 and/or HPLC detection of densely glycosylated S-proteins on the SARS-CoV-2 virion surface. In acute COVID-19 patients with HbA1c>6.0% without concurrent glycemia, we suggest repeated HbA1c testing after recovery. In our cohort, DM developed in 7.24% of all subjects without DM history in 52±2 weeks after discharge, which corresponds to 16.6% of the total number of patients with HbA1c >6.0% during follow-up. Therefore, we can assume that SARS-CoV-2 contributes to DM manifestation in subjects with possible previous CM disorders and a high risk of DM.
