# Correction: Glycated hemoglobin level dynamics in COVID-19 survivors: 12 months follow-up study after discharge from hospital

**DOI:** 10.1371/journal.pone.0302301

**Published:** 2024-04-11

**Authors:** The PLOS One Staff

In the Funding statement, the grant number from the funder the Ministry of Science and Higher Education of the Russian Federation is listed incorrectly. The correct grant number is: 075-15-2022-310 (20.04.2022).

The publisher apologizes for the error.
